# Association of Serum Neuron-Specific Enolase and C-Reactive Protein With Disease Location and Endoscopic Inflammation Degree in Patients With Crohn's Disease

**DOI:** 10.3389/fmed.2021.663920

**Published:** 2021-08-26

**Authors:** Rui-Xia Yang, Wei-Juan Song, Zhi-Qi Wu, Hemant Goyal, Hua-Guo Xu

**Affiliations:** ^1^Department of Laboratory Medicine, The First Affiliated Hospital of Nanjing Medical University, Nanjing, China; ^2^Department of Internal Medicine, Wright Center for Graduate Medical Education, Scranton, PA, United States

**Keywords:** NSE, CRP, crohn's disease, IBD, diagnosis

## Abstract

**Objective:** The objective of this study was to explore the association between serum markers neuron-specific enolase (NSE) and C-reactive protein (CRP) with intestinal lesion location and degree of inflammation in patients with Crohn's disease (CD).

**Design:** The levels of serum NSE, CRP, and fecal calprotectin (FC) in patients with CD were analyzed retrospectively. The severity of inflammatory lesions in the intestinal wall was accessed using the Simple Endoscopic Score for Crohn's disease (SES-CD).

**Results:** The levels of NSE in patients with CD were higher than those of healthy individuals (14.87 vs. 12.68 ng/ml, *P* < 0.001). The levels of CRP in patients with CD were higher than those of healthy individuals (12.30 vs. 3.40 mg/l, *P* < 0.001). The FC levels in patients with CD were higher than those of patients with non-inflammatory bowel disease (1,143.90 vs. 114.21 μg/g, *P* < 0.05). The levels of NSE in CD with ileal lesions and simultaneous ileal and colon lesions were significantly higher than those in patients with CD with colonic lesions. However, the CRP was higher in patients with colonic lesions than those with ileal lesions. The levels of NSE in patients with severe inflammation were higher than those in patients with moderate inflammation (15.95 vs. 13.89 ng/ml, *P* < 0.05). Similarly, the NSE levels in patients with CD with severe inflammation were higher than those in patients with CD with mild inflammation (15.95 vs. 13.53 ng/mL, *P* < 0.05). The levels of CRP in severe inflammation were higher than those in moderate inflammation (29.80 vs. 19.60 mg/l, *P* < 0.05). In addition, the CRP levels in severe inflammation were higher than those in mild inflammation (29.80 vs. 5.86 mg/l, *P* < 0.05). ROC curve analysis showed that when NSE was combined with CRP for distinguishing between patients with CD and those without CD, sensitivity increased to 80.41%, specificity increased to 74.66%, and a highest AUC was equal to 0.843.

**Conclusion:** Our study shows that serum NSE and CRP can be used to assess the severity of CD as well as the location of intestinal involvement. Therefore, NSE and CRP could be used as the non-invasive tests in detecting the location and severity of disease in patients with CD in daily routine practice.

## Introduction

Crohn's disease (CD) is a chronic inflammatory condition with transmural involvement of the gastrointestinal tract (GIT), a form of inflammatory bowel disease (IBD) (1, 2). CD can involve ileal (30%), ileocolonic (40%), and colon (30%) cases, unlike ulcerative colitis (UC) (3). Chronic active inflammation can lead to recurrent ulcers, intestinal stenosis, intestinal perforation, abdominal abscess, anal fistula, and colorectal cancer (4–6). Endoscopy is currently the main method to diagnose and estimate the disease severity, but a combination of clinical, laboratorial, endoscopic, and histological and sometimes radiological methods is required.

Multiple biomarkers such as serum calprotectin, albumin, and C-reactive protein (CRP) have been studied to estimate the disease severity in CD (7). Many studies have recently shown that fecal calprotectin (FC) can be used as a non-invasive biomarker of disease activity (8–10). However, FC might not be increased in patients with CD who predominantly have ileal involvement (11). In addition, the pretreatment process of stool samples is complicated, which will lead to low test repeatability.

Neuron-specific enolase (NSE) is a specific neuronal marker of differentiation. NSE is a protein localized in the cytoplasm of the neurons, red blood cells and platelets, etc. (12). It is involved in the glycolytic energy metabolism in the neurons and is often released from neurons as a response to injury. The levels of NSE have been found to be elevated in the many conditions including traumatic brain injury, post-cardiac arrest, and lung cancer. In fact, NSE is currently the most reliable tumor biomarker in the diagnosis, prognosis, and follow-up of small cell lung cancer (13). Busikova-Malenovska et al. (14) reported the localization of NSE in the neurons of the intestinal wall of five patients with CD using monoclonal antibodies against human-NSE. Here, we aimed to investigate the levels of NSE in patients with CD and further to evaluate their application in the diagnosis and disease severity.

CRP is an acute-phase biomarker with increased levels in some inflammatory conditions. CRP has also been used to differentiate functional bowel disorders such as irritable bowel syndrome from CD (15). However, studies have produced inconsistent results regarding the elevation in CRP levels in patients with active CD. Florin et al. found that 10% of active patients with CD (defined by CDAI >200) had normal CRP values. They further analyzed that patients with active disease but normal CRP levels had significantly more isolated ileal disease (16). No patients in active colonic CD had normal CRP levels. Another study found that elevated high-sensitivity CRP (hs-CRP) was associated with ileal (43.2%), colonic (70%), and ileocolonic (72.6%) diseases. The mean hs-CRP was also significantly higher in ileocolonic/colonic when compared to ileal disease (17). However, other studies did not find a significant association between CRP and disease location (18, 19).

In the present study, we have retrospectively explored the relationship of serum biomarkers NSE and CRP with the CD intestinal lesion location and degree of inflammation in an effort to provide a basis for their use in the diagnosis and treatment of CD.

## Materials and Methods

### Ethical Consideration

Ethics approval was obtained from the ethics committee of the First Affiliated Hospital of Nanjing Medical University (Protocol # 2020-SR-010, 23 March 2020). The study protocol conforms to the ethical guidelines of the 2013 Declaration of Helsinki as reflected in a prior approval by the institution's human research committee. Due to the retrospective nature of the study, informed consent was waived.

### Study Populations

The study cohort consisted of newly diagnosed patients with CD who were followed up at the First Affiliated Hospital of Nanjing Medical University between January 1, 2013, and October 31, 2018. The CD was diagnosed on the basis of patients' clinical manifestations, abdominal imaging, endoscopy findings, and pathology results. Exclusion criteria for both patients and healthy people included (1) those who take non-steroidal anti-inflammatory drugs and proton pump inhibitors, (2) alcoholics, (3) people with autoimmune diseases, (4) those who had a history of gastrointestinal surgery, (5) those with a history of liver and kidney diseases and a history of cardiovascular and cerebrovascular diseases, (6) pregnant women, and (7) cancer patients.

### Description of Variables

Patients' information including demographics, clinical presentation, duration of the disease, disease location, presence of perianal lesions, and serum levels of NSE were collected. The severity of inflammatory lesions in the intestinal wall was accessed using the Simple Endoscopic Score for Crohn's Disease (SES-CD) (20).

### Collection of Samples and Test Interpretation

A fasting (for 12–14 h) venous blood sample (around 3 ml) was collected from the subjects in vacuum inert separation gel conduit on the day before endoscopy. The upper serum was collected after centrifugation for 5 min at 3,000 r/min (centrifugal radius 17.49 cm). The CRP levels were assessed using a BN-II-specific protein analyzer and its matching reagents. The NSE levels were assessed using a Roche cobas e 602 electrochemiluminescence immunossay instrument and its matching reagents; the intra-/inter-assay CVs for NSE measurements were very small. All tests were strictly operated according to the instrument and reagent instructions. Each batch of tests was tested for low-value and high-value quality control. The FC was assessed with the standard enzyme-linked immunosorbent assay (ELISA) method produced by Bühlmann, Switzerland.

### Statistical Analysis

The IBM SPSS 27.0 and R version 3.6.3 software were used for the statistical analysis. The skewness coefficient and kurtosis coefficient are calculated to judge the distribution of data. The continuous variables which were non-normally distributed were expressed as the median and range. Differences in characteristics between groups were analyzed using the Kruskal–Wallis test with Dunn *post hoc* tests (for continuous variables, R package FSA), and the Bonferroni-adjusted *P*-value was reported. Dunn's Kruskal–Wallis multiple comparisons and *P*-values adjusted with the Bonferroni method were used for the comparison between the two groups. The receiver operating characteristic (ROC) curve was established to evaluate the NSE and/or CRP levels to distinguish the patients with and without CD. The ROC curves were plotted using the “pROC” package by R. In this analysis, patients with (case, encoded as 1) and without (control, encoded as 0) CD were considered as response and the NSE and/or CRP levels were predictors. A binormal smoothing was performed as the ROC approximation method. To analyze the relationship between different inflammation degrees, different lesion locations, NSE, and CRP, univariate multivariate multinomial logit regression analysis was performed. In this model, the different inflammation degrees and different lesion locations were considered as dependent variables, while the levels of NSE and CRP were considered as independent variables.

## Results

### Patients

From January 2013 to December 2018, a total of 291 confirmed cases of CD were included in the study. The control group consisted of 292 healthy people and 64 patients with non-inflammatory bowel disease (6 patients with intestinal obstruction, 16 patients with irritable bowel syndrome, 11 patients with intestinal polyps, and 31 patients without gastrointestinal disease). Two hundred and ninety-one patients with CD comprising 182 males aged 14–81 years (mean age 32.14 ± 13.27 years) and 109 females 14–77 years of age (mean age 43.25 ± 13.59 years) were included in the analysis. The data for 292 healthy individuals (182 males, mean age 33.45 ± 8.76 years and 110 females, mean age 38.72 ± 13.44 years) were collected. We also collected data for 64 patients with non-inflammatory bowel disease comprising 29 males (mean age 50.86 ± 18.90 years) and 35 females (mean age 51.46 ± 18.98 years) as the control during the same period. The main characteristics of the patients with CD are summarized in Table 1, as per the Montreal classification. The two groups did not show any significant differences with respect to age and gender.

**Table 1:** Baseline characteristics of patients with CD at the time of endoscopy.

	**Overall cohort (***n*** = 291)**
Sex (M/F)	182/109
Age (years), mean (range)	36.3 (14–81)
Weight change, *N* (%)	
Without weight change	204 (70)
Weight loss	87 (30)
Symptoms, *N* (%)	
Diarrhea only	51 (17)
Abdominal pain only	98 (34)
Diarrhea and abdominal pain	111 (38)
Others	31 (11)
Age (years), *N* (%)	
A1	7 (2.4)
A2	178 (61.2)
A3	106 (36.4)
Disease behavior, *N* (%)	
B1	113 (39)
B2	65 (22)
B3	113 (39)
CD location, *N* (%)	
L1	104 (36)
L2	56 (19)
L3	117 (40)
L4	14 (5)
History of perianal lesions, *N* (%)	102 (35)
Crissum abscess	50 (17)
Anal fistula	34 (12)
Others	18 (6)

*CD, Crohn's disease; F, female; M, male*.

The average CV for NSE measurements was 3.1% in 2018. A total of 245 patients had levels of serum CRP, and 53 patients underwent testing for FC at the time of admission. The NSE levels in patients with CD were higher than those in healthy individuals [14.87(6.10–51.66) ng/ml vs. 12.68(3.73–25.20) ng/ml] (Z = −7.030, *P* < 0.001). The CRP levels in patients with CD were higher than those in healthy individuals [12.30(0.16–112.00) mg/l vs. 3.40(0.22–24.80) mg/l] (Z = −8.429, *P* < 0.001). The FC levels in patients with CD were higher than those in patients with non-inflammatory bowel disease [1143.90(16.32–3403.64) μg/g vs. 114.21(3.15–5497.78) μg/g] (Z = −2.245, *P* < 0.05).

### Levels of NSE and CRP Based on the Intestinal Location of Crohn's Disease

**Figure 1:**
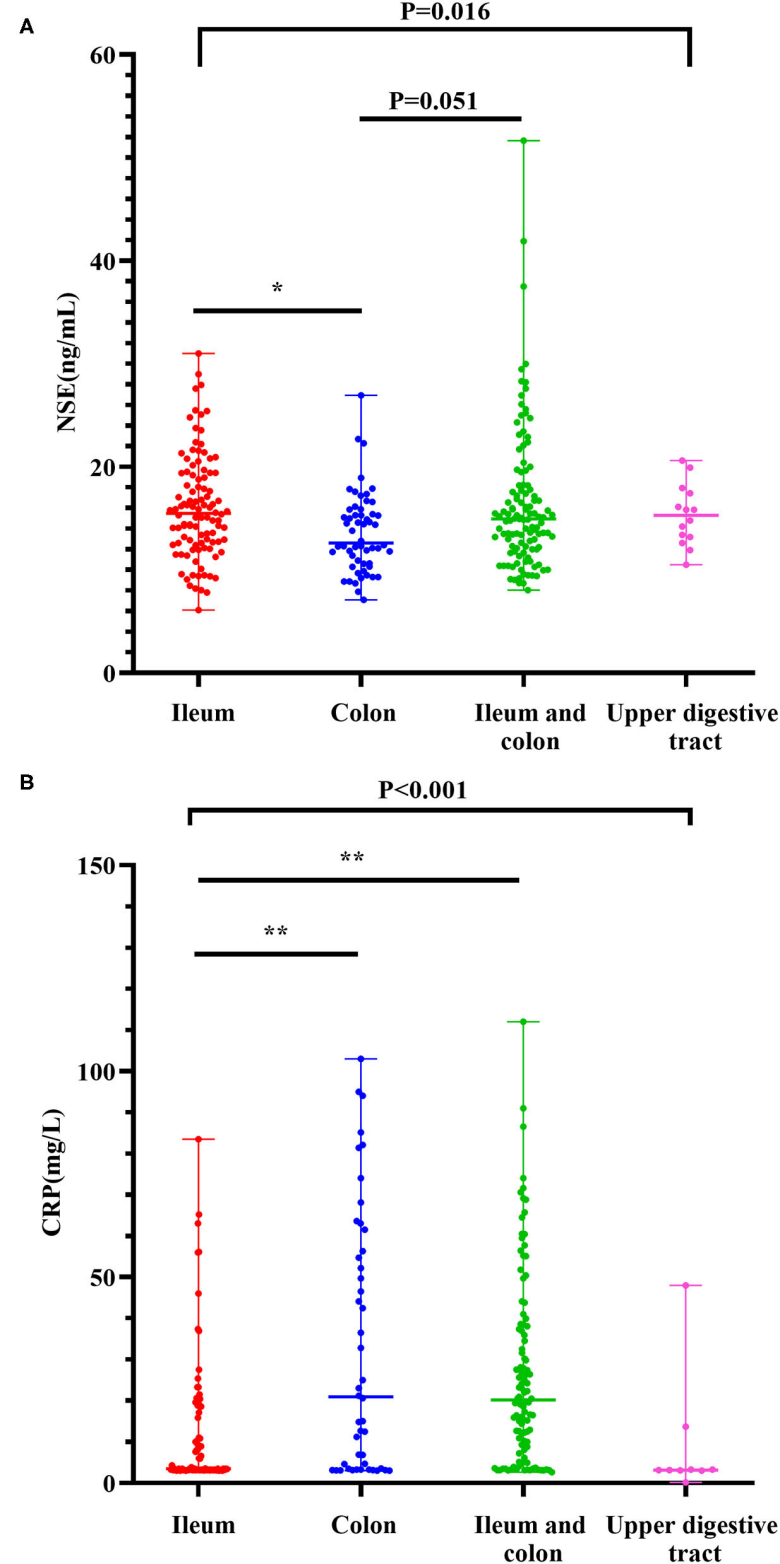
Levels of NSE as per the phenotype of Crohn's disease **(A)**. Levels of CRP as per the phenotype of Crohn's disease **(B)**. The boxplots show median, upper, and lower quartiles of the data; the whiskers indicate the 95% confidence interval of the values (*P < 0.05 and **P < 0.001).

Levels of NSE in CD with ileal lesions were higher than those in patients with CD with colon lesions [15.48(6.10–31.00) ng/ml vs. 12.61(7.10–26.95) ng/ml] (Z = −3.103, *P* = 0.012). The NSE levels in patients with ileocolonic lesions were higher than those with only colonic lesions [14.94(8.04–51.66) ng/ml vs. 12.61(7.10–26.95) ng/mL] (Z = −2.620, *P* = 0.051) (Figure 1A). The result of the multinomial logit model showed that between colonic lesions in patients with CD and ileal lesions in patients with CD, the increased NSE level was more common in patients with ileal lesions (OR = 0.32, *P* < 0.001), see in Table 2.

**Table 2:** Association between different inflammation degrees, different lesion locations and NSE and CRP levels.

		**log2(NSE)**		**log2(CRP)**	
		**OR (95% CI)**	***P***	**OR (95% CI)**	***P***
Lesion location					
	Colon/ileal lesion	0.32 (0.15–0.69)	<0.001	1.70 (1.34–2.16)	<0.001
	Ileocolonic lesion/ileal lesion	1.01 (0.57–1.79)	0.96	1.70 (1.40–2.07)	<0.001
	Upper gastrointestinal lesion/ileal lesion	0.90 (0.27–3.04)	0.87	0.64 (0.36–1.11)	0.11
Degree of inflammation					
	Moderate inflammation/mild inflammation	1.35 (0.50–3.63)	0.55	1.28 (0.95–1.72)	0.10
	Severe inflammation/mild inflammation	3.57 (1.36–9.39)	0.01	1.59 (1.17–2.17)	<0.001

The CRP levels in colonic patients with CD were higher than those in ileal patients with CD [20.90(3.01–103.00) mg/l vs. 3.44(3.01–83.50) mg/l] (Z = −4.251, *P* < 0.001). In addition, the levels of CRP in ileocolonic patients with CD were higher than those in ileal patients with CD [20.15(2.66–112.00) mg/l vs. 3.44(3.01–83.50) mg/l] (Z = −5.577, *P* < 0.001) (Figure 1B). The results of the multinomial logit model showed that between colonic lesions in patients with CD and ileal lesions in patients with CD, the increased CRP level was more common in patients with colon lesions (OR = 1.70, *P* < 0.001); between ileocolonic lesions in patients with CD and ileal lesions in patients with CD, the increased CRP level was more common in patients with ileocolonic lesions (OR = 1.70, *P* < 0.001) (see Table 2).

### The Association of the Levels of NSE and CRP With SES-CD

**Figure 2:**
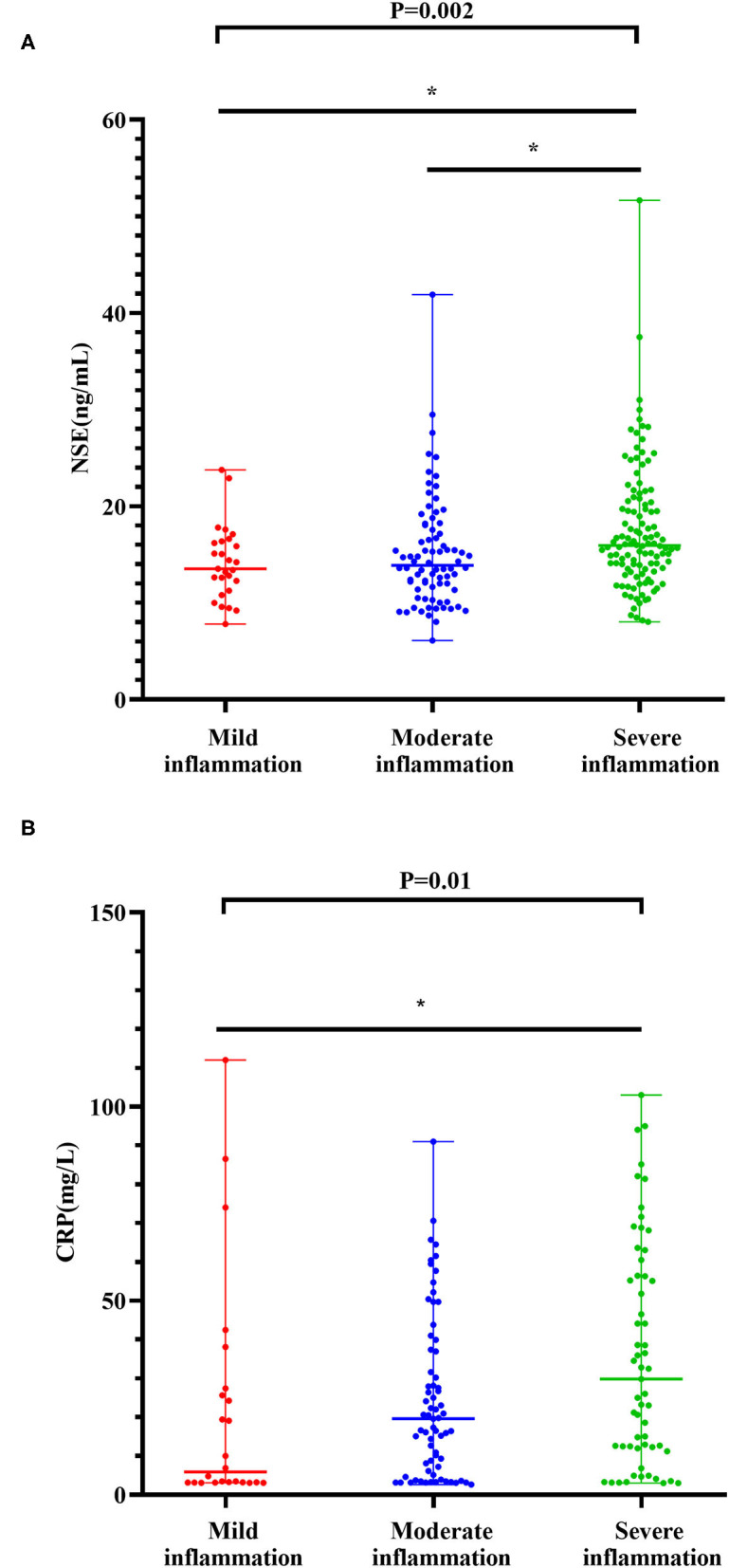
Levels of NSE with degrees of inflammation in the CD cohort **(A)**. Levels of CRP with degrees of inflammation in the CD cohort **(B)**. The boxplots show median, upper, and lower quartiles of the data; the whiskers indicate the 95% confidence interval of the values (*P < 0.05 and **P < 0.001).

The levels of NSE in patients with severe inflammation were higher than in patients with moderate inflammation [15.95(8.04–51.66) ng/ml vs. 13.89(6.10–41.90) ng/ml] (Z = −3.068, *P* = 0.006). Similarly, the levels of NSE in patients with CD with severe inflammation were higher than those in patients with mild inflammation [15.95(8.04–51.66) ng/ml vs. 13.53(7.80–23.77) ng/ml] (Z = −2.590, *P* = 0.029) (see Figure 2A). In addition, the results of the multinomial logit model showed that between patients with CD with severe inflammation and patients with CD with mild inflammation, the increased NSE level was more common in patients with severe inflammation (OR = 3.57, *P* < 0.05), see in Table 2.

The levels of CRP in severe inflammation were higher than those in moderate inflammation [29.80(3.01–103.00) mg/l vs. 19.60(2.66–91.00) mg/l] (Z = −1.942, *P* = 0.156). The levels of CRP in severe inflammation were higher than those in mild inflammation [29.80(3.01–103.00) mg/l vs. 5.86(3.10–112.00) mg/l] (Z = −2.916, *P* = 0.011) (see in Figure 2B). The results of the multinomial logit model showed that between patients with CD with severe inflammation and patients with CD with mild inflammation, the increased CRP level was more common in patients with severe inflammation (OR = 1.59, *P* < 0.001) (see in Table 2).

### Receiver-Operating Characteristic Analysis

**Figure 3:**
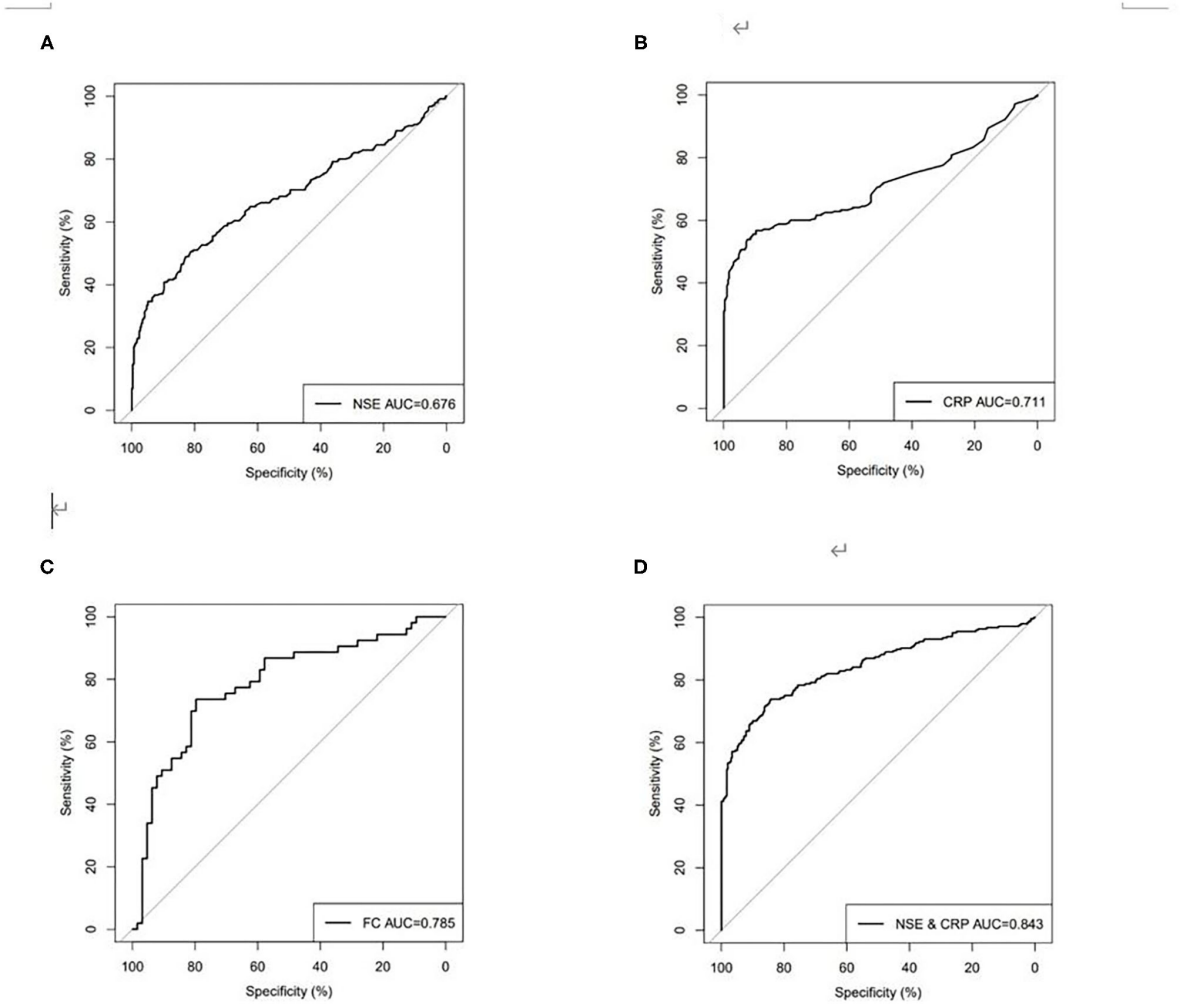
Receiver operating characteristics (ROC) curves for **(A)** NSE, **(B)** CRP, **(C)** FC, **(D)** combined NSE and CRP for discriminating between patients with CD and healthy people.

Using ROC analysis, the optimal cutoff point for the serum NSE level in order to distinguish patients with CD from those without CD was 15.06 ng/ml, with a sensitivity of 48.98%, a specificity of 82.88%, and the highest area under the curve (AUC) equal to 0.676 (0.629–0.723, *P* < 0.001). The optimal cutoff point for the serum CRP level in order to distinguish patients with CD from those without CD was 7.93 mg/l, with a sensitivity of 56.73%, a specificity of 89.73%, and a highest AUC equal to 0.711 (0.664–0.758, *P* < 0.001). The optimal cutoff point for FC in order to distinguish patients with CD from those without CD was 752.40 μg/g, with a sensitivity of 73.58%, a specificity of 79.69%, an accuracy of 76.92%, and the highest AUC equal to 0.785 (0.700–0.871, *P* < 0.001). However, if the current agreed standard normal value of FC (50.00 μg/g) is used as the cutoff point, the sensitivity was 88.68%, the specificity was 34.38%, and the accuracy was only 58.97%. ROC curve analysis showed that when NSE was combined with CRP for distinguishing between patients with CD and those without CD, sensitivity increased to 80.41%, specificity increased to 74.66%, and the highest AUC was equal to 0.843 (0.808–0.878, *P* < 0.001) (Table 3, Figure 3), and spearman rank correlation showed that there was no correlation between NSE and CRP(r = 0.025, *P* = 0.567).

**Table 3:** Sen, Spe, Acc, PPV, and NPV for serum NSE, CRP, and FC in discriminating between patients with CD and healthy people.

	**Sen (%)**	**Spe (%)**	**Acc (%)**	**PPV (%)**	**NPV (%)**
NSE (in ng/mL cutoff 15.06)	48.98	82.88	67.41	70.59	65.94
CRP (in mg/L cutoff 7.93)	56.73	89.73	74.67	82.25	71.20
FC (in μg/g cutoff 752.40)	73.58	79.69	76.92	75.00	78.46
NSE+CRP	80.41	74.66	77.28	72.69	81.95

*CD, Crohn's disease; NSE, neuron-specific enolase; CRP, C-reactive protein; FC, fecal calprotectin; Sen, sensitivity; Spe, specificity; Acc, accuracy; PPV, positive predictive value; NPV, negative predictive value*.

## Discussion

In this study, we found that levels of NSE and CRP in patients with CD were significantly higher than those of healthy people. Neuroproliferation has been shown to be a feature of CD with the use of NSE (21). However, our study is the first clinical study to evaluate the utility of NSE in daily routine practice in patients with CD. In addition, our study shows that high CRP levels could be useful as an adjunct in the diagnosis of CD, which is consistent with the results of most previous studies.

We further performed the analysis based on the intestinal location of the disease. We found that levels of NSE in patients with CD with ileal lesions were higher than those in patients with CD with colon lesions. NSE levels in patients with ileocolonic lesions were higher than those with only colonic lesions. Most studies have shown that serological markers such as CRP can aid in diagnosing CD and have refined the diagnosis to the site of the damage (22). The mechanism of the different concentrations of NSE in different damaged digestive tract sites is not well understood. We suspect that this difference may be due to the uneven distribution of intestinal neurons in the ileum and colon. Some relevant studies have revealed similar neuronal and ganglionic densities and neurochemical profiles in the human distal colon and rectum (23). However, the comparison of neuronal and ganglionic densities and neurochemical profiles in the human ileum and colon have not been reported.

Bourgonje et al. (24) recently reported that inflammatory biomarkers [serum amyloid A (SAA), Eotaxin-1, IL-6, IL-8, IL-17A, and TNF-α] showed better prediction of IBD disease activity than routine measures (CRP, fecal calprotectin, and HBI/SCCAI scores) and demonstrated that the combination of SAA, IL-6, IL-8, and Eotaxin-1 could reliably predict endoscopic disease activity in IBD, which contributed to establishing an immunology-based prediction model for the endoscopic mucosal status in IBD. In our study, in addition to the abovementioned diagnostic significance of NSE and CRP in the different parts of the digestive tract, we also found that levels of NSE and CRP varied with the amount of inflammation in the intestines. Patients with CD with severe inflammation have higher levels of NSE and CRP than patients with moderate and mild inflammation. Therefore, NSE and CRP may be of added value as inflammatory biomarkers in monitoring disease activity in CD. Studies have shown that high CRP levels often indicate poor prognosis and increased incidence of complications in patients with CD (25). However, higher inflammatory biomarker levels were not observed in some patients who did not get symptomatic relief (26), which indicated that the degree of inflammation does not fully reflect the prognosis of the disease. Margolis et al. (27) found that the severity of intestinal inflammation is associated with the density of the enteric innervation in mice. The authors postulated that the abnormalities in enteric nervous system development might contribute to the pathogenesis of IBD. There have been a number of previous clinical reports of increased numbers of enteric neurons in the inflamed regions of the bowel in IBD (28, 29), as well as studies that have shown an association of neuronal genes (such as LRRK2 and Ninjurin2) with the increased risk of IBD (30, 31). However, it is not yet clear how enteric neuronal density affects the severity of intestinal inflammation. It has been postulated that effectors of both innate and acquired immunity express receptors for neurotransmitters/neuromodulators. Some immune cells express nicotinic receptor subunits and that nicotinic stimulation is anti-inflammatory, which probably accounts for intestinal anti-inflammatory properties of vagus nerve stimulation (32, 33). Moreover, this is the first study reporting the increase in the serum NSE concentration with an increase in intestinal inflammation in patients with CD, laying the foundation for use of NSE for CD diagnosis.

Determination of FC levels has been used as a good screening method in the diagnosis as well for the evaluation of acute exacerbation of CD (9). Calprotectin is a major protein found in the cytosol of inflammatory cells (34). FC is an effective biomarker in the diagnosis and treatment of patients with CD. However, the collection of fecal samples is not easy to standardize, which affects the detection results. Moreover, the detection process is cumbersome and time-consuming, and there are many influencing factors in the detection process, so the results vary quite greatly. Our results have shown that the combined use of NSE and CRP is comparable to FC on the accuracy of CD diagnosis. Moreover, the collection of blood samples is easy to be standardized, and the detection process of NSE and CRP is automated, so the detection results have better repeatability and reproducibility and easier to carry out in clinical practice.

We would also like to note that our study has a small sample size, an inherent shortcoming of retrospective cohort analysis. Not all patients in the CD cohort underwent serum NSE and CRP testing at the same time. Only 53 patients underwent FC testing, as this test was not available in our hospital before 2018. However, the results of our study are very important, as we have presented NSE as a potential marker for diagnosis of CD as well as its utility in the determination of the degree of intestinal inflammation. We have tried to expand the armamentarium of the previously available biomarkers that reflect the degree of inflammation.

Crohn's disease imposes a huge financial burden on the affected patients and requires early diagnosis and aggressive management to reduce complications. Although endoscopy is a traditional invasive method of evaluation of the diagnosis and monitoring of the disease, the majority of the patients can avoid the use of endoscopy with the help of effective biomarkers (35, 36). Therefore, strategies to select the patients with CD who would benefit most from endoscopy are of current interest. Serum biomarkers provide a convenient, rapid, and non-invasive method for clinical diagnosis and disease activity monitoring. Our study shows that serum NSE and CRP can be used to assess the severity of CD as well as the location of intestinal involvement. Therefore, NSE and CRP could be used as non-invasive tests to detect the location and severity of disease in patients with CD in daily routine practice. In addition, this study will pave the way for further prospective studies for NSE use as a non-invasive biomarker for the diagnosis and disease monitoring in patients with CD.

## Data Availability Statement

The raw data supporting the conclusions of this article will be made available by the authors, without undue reservation.

## Ethics Statement

The studies involving human participants were reviewed and approved by Ethics approval was obtained from the ethics committee of the First Affiliated Hospital of Nanjing Medical University (Protocol # 2020-SR-010, 23 March 2020). The patients/participants provided their written informed consent to participate in this study. Written informed consent was obtained from the individual(s) for the publication of any potentially identifiable images or data included in this article.

## Author Contributions

All authors listed have made a substantial, direct and intellectual contribution to the work, and approved it for publication.

## Conflict of Interest

The authors declare that the research was conducted in the absence of any commercial or financial relationships that could be construed as a potential conflict of interest.

## Publisher's Note

All claims expressed in this article are solely those of the authors and do not necessarily represent those of their affiliated organizations, or those of the publisher, the editors and the reviewers. Any product that may be evaluated in this article, or claim that may be made by its manufacturer, is not guaranteed or endorsed by the publisher.
